# Miscellaneous

**Published:** 1892-03

**Authors:** 


### MISCELLANEOUS.

### HOW TO HAVE A BRIGHT LAMP.

In these days when lamps are used so much the care of them is quite an important matter. If the lamps be good and have proper attention one cannot wish for a more satisfactory light, but if badly cared for they will be a source of much discomfort. The great secret of having lamps in good working order is to keep them clean and to use good oil. Have a regular place and time for cleaning the lamps. Put a folded newspaper on the table, so that any stray bits of burnt wick or drops of oil may fall upon it. Wash and wipe the chimneys and shades. Now take off all loose parts of the burner, washing them in hot soap suds and wiping with a clean, soft cloth. Trim the wicks and turn them quite low. With a soft, wet cloth, well soaked, wipe the burner thoroughly, working the cloth as much as possible inside the burner, to get off every particle of the charred wick. Now fill the lamps within about one inch of the top and wipe with a damp towel and then a dry one. Adjust all the parts and return them to their proper places. Whenever a new wick is required in a lamp, wash and scald the burner before putting in the wick. With a student lamp the receptacle for waste oil, which is screwed on the bottom of the burner, should be taken off at least once a week and washed. Sometimes a wick will get very dark and dirty before it is half consumed. It is not economy to try to burn it; replace it with a fresh one. The trouble and expense are slight and the increase in clearness and brilliancy will repay the extra care. When a lamp is lighted it should not at once be turned up to the full height; wait until the chimney is heated. Beautiful shades are often cracked or broken by having the hot chimneys rest against them. Now, when lighting a lamp, be careful that the chimney is set perfectly straight and does not touch the shade at any point. The shade should be placed on the lamp as soon as it is lighted, that it may heat gradually,

### THE "DOUBLEGANGER."

One of the stories collected by Mr. Stead is that of a "double" related by Dr. F. R. Lees, a well known English temperance reformer. I had, he says, left Leeds for the Isle of Jersey (though my dear wife was only just recovering from a nervous fever) to fulfill an important engagement. On a Good Friday myself and a party of friends in several carriages drove round a large portion of the island, coming back to St. Heliers from Boulay Bay, taking tea about 7 o'clock at Captain ———'s villa. The party broke up about 10 o'clock, and the weather being fine and warm I walked to the house of a banker who entertained me. Naturally my evening thoughts reverted to my home and after reading a few verses in my Testament I walked about the room until nearly 11, thinking of my wife and breathing the prayer "God bless you." I might not have recalled all the circumstances, save for the letter I received by the next post from her, with the query put in: "Tell me what you were doing within a few minutes of 11 o'clock on Friday evening? I will tell you in my next why I ask; for something happened to me." In the middle of the week the letter came, and these words in it: "I had just awoke from a slight repose, when I saw you in your night dress bend over me and uttering the words, 'God bless you!' I seemed also to feel your breath as you kissed me. I felt no alarm, but comforted, went off into a gentle sleep, and have been better ever since." I replied that this was the exact representation of my mind and words.

### A LETTER BY COTTON MATHER.

The following is the full text of a letter written by Cotton Mather to John Higginson, dated Boston, Mass., September 3, 1682:

To Ye Aged and Beloved John Higginson: There be now at sea a skipper (for our friend Esasias Holdcroft, of London, did advise me by the last packet that it would sail some time in August) called Ye Welcome, R. Green was master, which has aboard a hundred or more of ye heretics and malignants called Quakers, with W. Penn, who is ye scamp at ye head of them. Ye general court has accordingly given secret orders to Master Malachi Huxtell, of ye brig Porpois, to waylay ye said Welcome as near ye Coast of Cod as may be and make captives of ye Penn and his ungodly crew, so that ye Lord may be glorified and not mocked on ye soil of this new country with ye heathen worshippe of these people. Much spoil can be made by selling ye whole lot to Barbadoes, where slaves fetch good prices in rumme and sugar, and we shall not only do ye Lord great service by punishing ye wicked, but shall make gayne for his ministers and people. Yours in the bowels of Christ. "COTTON MATHER."

The letter is written in the peculiar style of phraseology common to the time, and is of interest from the fact that it is concerning a man who succeeded in establishing a colony of people in this country from which developed a powerful religious sect, whose love of country, patriotism and high sense of morality have been the bulwarks of our civilization.

---

WASHING FRUIT BEFORE EATING IT.—The following curious instance is reported by M. Schnirer of the ease with which consumption germs may be disseminated. While at work one day in the laboratory of Weichselbaum, he sent for some grapes to eat. The fruit had been kept for some time in a basket outside the lavatory, and was covered with dust, so that the water in which it was washed was black. On examining it he reflected that, inasmuch as the neighboring street was traversed by consumptive patients going to the clinic, the dust probably was charged with tubercle bacilli. To settle this, M. Schnirer injected into three guinea pigs ten cub. centim. of the water in which the grapes had been washed. One animal died in two days from peritonitis, the two others died on the forty-eighth and fifty-eighth days respectively, presenting marked tuberculous lesions, especially at the place of injection. The water in which the grapes had been washed was taken from the faucet, and the glass containing it had been sterilised ; neither the boy who had bought the grapes, nor the merchant who had sold them, was consumptive. The cause of infection was, beyond doubt, the dust on the grapes. This experiment illustrates the danger arising from the dissemination of desiccated tuberculous sputa in the air.

IN a steel mill at Newburn, England, is a machine that will shear an ingot of steel thirty inches in width and twelve inches in thickness. A mighty foot holds the ingot in place, and the knife descends and snips off a piece as a boy cuts candy. Hydraulic power is used, and the cut can be made in about three seconds.

IN DYSENTERY, as in diarrhœa, people generally consider mild attacks as trifling, and either disregard them at first or but indifferently treat them with domestic remedies. Simple treatment properly applied would in many cases prove successful, but the trouble is, it is not, as a rule, properly applied. Many persons, when they feel an attack of dysentery coming on, resort to stimulants, and especially to brandy ; others seek obliging druggists, and from them obtain medicines. Neither the former nor the latter class are very often benefited by the methods which they employ. Stimulants are very rarely needed in dysentery, and in no instance are they allowable unless sanctioned by a physician.

TO KILL COCKROACHES.—A housekeeper who was recommended to try cucumber peeling as a remedy for cockroaches, strewed the floor with pieces of the peel cut not very thin, and watched the sequel. The pests covered the peel in a short time, so that it could not be seen, so voraciously were they engaged in sucking the poisonous moisture. The second night this was tried the number of cockroaches was reduced to a quarter and none were left alive on the third night.

"JAMES, how much is four, plus eight, plus one ?" asked the teacher. "Don't know," said James. "Well, suppose I gave four apples, to Harry, eight apples to Charlie, and one to you. What would it be ?" "A cold day for me," whimpered James.

HINTS FOR NURSES.—Sick people don't like to be stared at. They are morbidly sensitive. To look surprised at the change sickness has wrought is annoying, and, worse than that, it is disheartening, and makes invalids imagine their case to be worse than it is. Therefore, don't stare at a sick person. And don't stand at the back of the head of the bed to make him turn his eyes round to see you. Always sit by the bedside, for the patient feels more at rest than if you stand up tall before him. And don't whisper ; don't talk in a low voice ; don't follow the doctor or a caller out into the next room. The invalid will be absolutely certain that you are discussing him. Don't wear garments that rustle or are made of rough cloth to come in contact with hands made tender by sickness, and don't wear creaking boots or thick-soled boots.

